# Hexavalent Chromium Removal from Industrial Wastewater by Adsorption and Reduction onto Cationic Cellulose Nanocrystals

**DOI:** 10.3390/nano12234172

**Published:** 2022-11-24

**Authors:** Francisco de Borja Ojembarrena, Hassan Sammaraie, Cristina Campano, Angeles Blanco, Noemi Merayo, Carlos Negro

**Affiliations:** 1 Department of Chemical Engineering and Materials, Complutense University of Madrid, Avda. Complutense s/n, 28040 Madrid, Spain; 2 Department of Microbial and Plant Biotechnology, Center for Biological Research Margarita Salas (CIB-CSIC), 28040 Madrid, Spain; 3 Department of Mechanical, Chemical and Industrial Design Engineering, ETSIDI, Polytechnic University of Madrid, Ronda de Valencia 3, 28012 Madrid, Spain

**Keywords:** cationic cellulose nanocrystals, hexavalent chromium, adsorption, wastewater treatment, cationization process

## Abstract

Cationic cellulose nanocrystals (CCNC) are lignocellulosic bio-nanomaterials that present large, specific areas rich with active surface cationic groups. This study shows the adsorption removal of hexavalent chromium (Cr(VI)) from industrial wastewaters by the CCNC. The CCNC were synthetized through periodate oxidation and Girard’s reagent-T cationization. The high value of CCNCs cationic groups and anionic demand reveal probable nanocrystal-Cr(VI) attraction. Adsorption was performed with synthetic Cr(VI) water at different pH, dosage, Cr(VI) concentration and temperature. Fast removal of Cr(VI) was found while operating at pH 3 and 100 mg·L^−1^ of dosage. Nevertheless, a first slower complete removal of chromium was achieved by a lower CCNC dosage (40 mg·L^−1^). Cr(VI) was fully converted by CCNC into less-toxic trivalent species, kept mainly attached to the material surface. The maximum adsorption capacity was 44 mg·g^−1^. Two mechanisms were found for low chromium concentrations (Pseudo-first and pseudo-second kinetic models and continuous growth multi-step intraparticle) and for high concentrations (Elovich model and sequential fast growth-plateau-slow growth intraparticle steps). The Sips model was the best-fitting isotherm. Isotherm thermodynamic analysis indicated a dominant physical sorption. The Arrhenius equation revealed an activation energy between physical and chemical adsorption. CCNC application at selected conditions in industrial wastewater achieved a legal discharge limit of 40 min.

## 1. Introduction

The presence of chromium in water effluents is related to several environmental concerns since it is cytotoxic and carcinogen to a wide range of species, along with other acute affections [1,2,3]. Chromium is typically present as hexavalent and trivalent species, although the environmental impact associated with chromium presence would vary depending on the species: while hexavalent is highly toxic, bioaccumulable and persistent in nature, even at low concentrations, the trivalent one shows less cytotoxicity [4]. The environmental effects of hexavalent chromium spillage would be present for years without adequate prevention due to its low biodegradability, adsorption onto the soil and the rapid uptake by plant cells [5]. Industrial wastewaters are the main sources of hexavalent chromium, including effluents from tanning, electroplating, wood preservation, cement, paint, stainless steel and metallurgical industries [6,7]. The maximum acceptable concentration of hexavalent chromium in water, according to the USEPA, is 0.1 mg·L^−1^, which reinforces the importance of minimizing its discharge to the environment [8]. 

Many technologies have been adopted to remove hexavalent chromium from effluents, but physical-chemical treatments are the most common. These processes show a great performance stability, allowing compliance with the required final quality in the effluents during their lifetime with minimal variations, which is desirable by the interested industries. Some of them include membrane treatments, adsorption, ion exchange, coagulation-flocculation, chemical precipitation and electrochemical processes [9]. Among these treatments, adsorption is widely implemented as an end-of-pipe treatment process for low-concentrated effluents because it is inexpensive, simple to install and manage, highly effective and its operation shows a low environmental impact. For these reasons, this process reaches great operating results in terms of technical and economic feasibility to prevent chromium contamination [10]. Several adsorbents have been used to successfully remove hexavalent chromium from water, varying from the most common activated carbons and zeolites and their varieties to many types of polymeric, mineral, organic, waste, or composite materials and nanomaterials [11,12]. Nowadays, the demand for cost-effective green sustainable adsorbents based on natural resources is increasing, but achieving high removal efficiencies is still a challenge. Cellulose meets most of these requirements, being a low-cost, renewable, non-toxic, biodegradable and the most abundant material, which could be obtained from lignocellulosic waste material. As well, this polymer is easily functionalized and chemically and physically modified to obtain a wide variety of cellulose-based materials [13]. Some certain cellulosic materials have been widely used as hexavalent chromium adsorbents, mainly in the form of raw lignocellulosic waste materials (including bagasses, fruit peels and wastes), composites with other polymers (such as polyethylenediamine and other polyamines) and inorganic materials (such as hydroxyapatite and hydrotalcite) or even as a support for highly active iron species as reducing agents (such as Fe_3_O_4_ or nano-zero valent iron (n-ZVI)) [14]. Nevertheless, among these common modification technologies, there is a lack of knowledge in the application of cationization techniques to celluloses, even when this modification would convert cellulose surfaces into attractive for anionic hexavalent chromium species. This interaction would facilitate the adsorption of these species, as expected by the adsorption results obtained through quaternary ammonium cations-modified montmorillonite by Yang et al. [15]. Moreover, higher fibrillation could lead to a higher adsorption capacity, which would increase the efficiency of the process. Nanocelluloses have one or more dimensions on the nano-sized scale [16] and present interesting properties, such as low density, high aspect ratio and mechanical strength and a large specific area, which are linked to good adsorbent materials. Besides the possibility of surface modification to promote active groups, these materials are suitable for adsorbing almost any kind of pollutant from wastewater reaching high yields [17]. 

Different types of nanocelluloses have been used for hexavalent chromium adsorption from wastewater. Cellulose nanofibers (CNFs) and nanocrystals (CNCs) from lignocellulosic sources and bacterial cellulose (BC) and their modifications have shown their capacity to attract hexavalent chromium anions. Yang, et al. [18] developed a hybrid structure based on BC coupled to poly(m-phenylenediamine) in nanoparticles with a total adsorption capacity of 434.78 mg·g^−1^. The hydrogel composite of chitosan/CNC grafted with carbon dots produced by Zeng, et al. [19] achieved 217.8 mg·g^−1^ of chromium adsorption capacity and could be used for quantitative detection of Cr(VI) up to 0.04 μg·L^−1^ thanks to the change in fluorescence of the material. The authors of this study developed a first approach in the application of nanocellulosic materials by studying the hexavalent adsorption onto hydrophobically modified CNF, reaching 70.38 mg·g^−1^ of adsorption capacity [20]. Even though the CNF showed proper characteristics to fix hexavalent chromium, an increase in the hexavalent chromium adsorption kinetics would be expected when other kinds of surface modifications are applied to nanocellulose, such as the cationization of CNCs. The generation of cationic charges on the surface of CNCs could increase the attractive interaction of the dissolved anionic hexavalent chromium species with the cellulosic adsorbent. In fact, the modification to generate quaternary ammonium cations on the surface of cellulose while reaching nano-scale cellulose crystals was previously achieved by Yang and van de Ven [21]. Unlike other surface modifications, such as citric acid-incorporated CNFs [22], the application of cationized CNCs has not been reported yet. There is a necessity for proof research in this field to generate new knowledge and lead further investigations. Together with the need for novel adsorbent materials, there is a tendency to focus on the direct adsorption of hexavalent chromium onto different materials without any change in chromium species. This fact implies the need to manage the hexavalent chromium-concentrated wash water counter currently and the spent adsorbent, which can be expensive and hazardous. With a view on the objective of reducing hexavalent chromium on the adsorbent, the production of a cellulose-based nanomaterial with reducing properties is desirable. As well, the obtention of this adsorbent-reducing nanocellulose in simple steps without the need for further materials for composites is also intended to ease the scale-up process. [23]. The current study is focused on the search for nanosized cellulosic materials that cover these mentioned requirements to fulfill with a seen necessity due to the reduced information about this topic. 

The aim of this study is the synthesis of cationized cellulose nanocrystals (CCNC) and the optimization of hexavalent chromium adsorption operating conditions using this nanomaterial. To the best of our knowledge, this CCNC material has not been applied in the adsorption-reduction treatment of hexavalent chromium in the previous bibliography. Its application supposes an advance in the search for the implementation of sustainable adsorbents for the removal of this pollutant. Our hypothesis is that CCNC, new lignocellulosic green nanoadsorbents with strong adsorptive properties, could be able to efficiently attract hexavalent chromium anions and instantly reduce them into less toxic trivalent chromium, a fact that has not been covered yet in the literature. The most relevant operating conditions, such as pH, adsorbent dosage, chromium concentration, temperature and contact time, were evaluated through laboratory batch adsorption. The obtained kinetic and isotherm data were analyzed to reach the optimal operating conditions. Data were adjusted to various mathematical models to reveal the adsorbate-adsorbent surface interaction mechanism. To facilitate the final implementation of the CCNC for industrial applications, this adsorbent was tested with urban wastewater samples from a wastewater treatment plant (WWTP) that received tannery industry effluents. Relevant and novel results in the adsorptive removal of hexavalent chromium in real industrial wastewaters through the use of CCNC were obtained in this study.

## 2. Materials and Methods

### 2.1. Materials

Commercial cotton linters supplied by Sigma Aldrich were utilized as a cellulose source to produce CCNC. The chemicals applied in the different steps of the synthesis and characterization processes were sodium (meta)periodate, Girard’s Reagent-T ((2-hydrazinyl-2-oxoethyl)-trimethylazanium chloride, GT), ethylene glycol, hydroxylamine hydrochloride, silver nitrate, potassium peroxodisulfate, sodium chloride and hydrochloric acid (37% *v*/*v*) which were supplied by Sigma Aldrich (St. Louis, MO, USA) and sodium hydroxide pellets supplied by Panreac (Barcelona, Spain). All the purchased chemicals were of analytical grade. 

Standard solutions of hexavalent chromium (50 mg·L^−1^) were used as calibration standards for the spectrophotometric analysis supplied by Hach. Hexavalent chromium in wastewater samples was analyzed following Standard Method 3500-Cr-B [24] through the analytical reagent kits supplied by Macherey Nagel (Düren, Germany). Poly(diallyldimethylammonium chloride) (PDADMAC) and polyethylenesulfonate (PesNA) standard solutions with 2.5·10^−4^ eq·L^−1^ of concentration were applied as titration reagents to determine cationic and anionic demand. 

### 2.2. CCNC Synthesis

**Figure 1:**
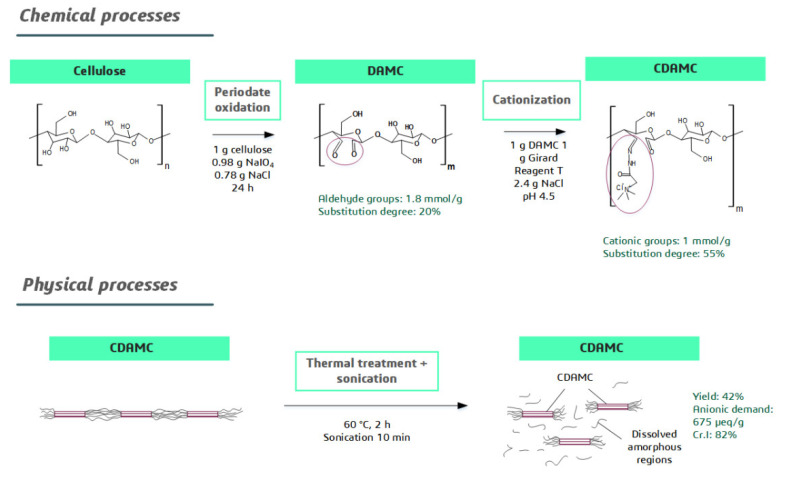
Graphical scheme of CCNC synthesis steps.

The preparation of CCNC was performed following the methodology developed by Yang and van de Ven [21]. The overall process consists of an initial dialdehyde formation reaction on cellulose to produce dialdehyde-modified cellulose (DAMC), followed by a Schiff-base reaction to synthetize cationic DAMC (CDAMC) and the last step involves heating and sonication treatments to produce nanosized cationic cellulose in form of cationic cellulose nanocrystals (CCNC). The synthesis steps are explained in the scheme presented in Figure 1. 

#### 2.2.1. Dialdehyde Formation Reaction

First, NaIO_4_ (0.98 g NaIO_4_·g^−1^ dried cellulose) and NaCl (0.78 g NaCl·g^−1^ d.c.) were added and once dissolved in water, cotton linters were dispersed in the sample (14.93 g d.c.·L^−1^). This photosensitive reaction was kept stirring in the dark for 24 h. After 24 h, ethylene glycol (1 mL·g^−1^ d.c.) was added to quench the non-reacted periodate. The final DAMC suspension was filtered and rinsed thoroughly.

#### 2.2.2. Surface Cationization Reaction

The GT reagent (1 g GT·g^−1^ d.c.) and NaCl (2.4 g NaCl·g^−1^ d.c.) were dissolved in water and then, the rinsed DAMC was suspended in the solution after modifying the pH to 4.5 with HCl. The suspension was stirred for 24 h and after that, the final CDAMC was filtered and washed.

#### 2.2.3. Synthesis of Nanocellulose

The filtered and washed CDAMC was diluted up to 1% (*w*/*w*) of consistency and kept under intense stirring at 60 °C for 2 h. The suspension was then sonicated for 10 min with an Ultrasonic Processor UP200H supplied by Hielscher (Germany). The CNCC suspension was purified by centrifugation at 5000 rpm for 10 min, separating the non-fibrillated fraction from the nanosized cellulose. 

### 2.3. CCNC Characterization

The physical-chemical characterization of CCNC involves common analytical parameters for celluloses and nanocelluloses. The anionic demand determination consists of a titration in which the charge of a colloidal suspension of a cationic cellulosic material is continuously measured. Once determined the charge of the suspension in mV, a standard solution of a well-known concentration of polyelectrolyte with the opposite (in this case, anionic) charge of the cationic cellulosic material is added. This anionic polyelectrolyte compensates the corresponding charge of the material until reaching the contrary sign of the charge on the solution, and the excess of the cationic polyelectrolyte added to the solution is back-titrated with the standard solution of cationic electrolyte until reaching neutral charge of the suspension (0 mV). Once reached, the obtained volume of cationic polyelectrolyte added to the cellulosic material allows the calculation of total charges through the normality of the standard solution and anionic demand can be calculated by dividing per unit of added mass of cellulose [25]. The anionic demand was determined through colloidal titration with a 2.5·10^−4^ eq·L^−1^ standard solution of PesNA using a Charge Analyzing system supplied by AFG Analytic GmbH. The determination of suspension consistency was evaluated as indicated by Balea, et al. [26]. Briefly, this method is based on the determination of the dried content of a cellulose sample after an overnight drying at 60 °C. The process yield, aldehyde content and cationic groups were measured through the methodology established by Campano, et al. [27]. The process yield consists of the percentage of solid sample maintained in the final suspension produced from the initial solid cellulose source. The aldehyde content of DAMC was measured through its titration with hydroxylamine hydrochloride. It reacts with aldehyde groups through Schiff base reaction with DAMC, producing hydrochloric acid. The produced acid is titrated by a standard solution of sodium hydroxide. The number of cationic groups is measured by conductimetric titration with AgNO_3_ 10 mM solution. Crystallinity index (Cr·I) was measured as established by Campano, et al. [28]. Summarized, XRD patterns were obtained with a Philips X’Pert MPD X-ray (Netherlands) diffractometer with an autodivergent slit fitted with a graphite monochromator applying CuKα working at 45 kV and 40 mA. The patterns were analyzed between 3 and 40° and the crystallinity index could be established by the Segal’s method with the values of intensity at the 002 interference (2Θ = 22.5°) and the amorphous scatter (2Θ = 18°). The weight content of carbon, nitrogen and hydrogen was measured through a LECO CHNS-932 elemental analyzer by the combustion of samples at 970 °C.

### 2.4. Batch Adsorption Experiments

The experimental procedure of batch adsorption tests was developed as indicated by Ojembarrena et al. [20]. Briefly, 100 mL synthetic wastewater samples are stirred under controlled operating conditions: contact time (up to 72 h), pH (3–9), chromium concentration (0.1–70 mg·L^−1^) and adsorbent dosage (10–400 mg·L^−1^). Blank and treated samples were placed in each experiment under the same conditions to assess the time evolution of the untreated sample and to be used as a control of possible environmental modifications in the samples. The temperature was controlled and monitored during the experiments. This parameter was also modified between 17 °C and 56 °C to evaluate its effect on adsorption. Samples were taken, filtered (0.45 μm) and chromium concentration was measured three times per water sample following the indications of the Standard Method 3500-Cr-B [24] at different contact times to achieve kinetic data. The last measured samples while varying initial chromium concentration corresponds to the equilibrium data applied to adjust isotherm models. 

The determination of trivalent chromium on the CCNC of the treated samples was also performed through spectrophotometry. This determination is based on the subtraction of the total chromium content (as hexavalent chromium specie) minus the hexavalent chromium concentration of the evaluated sample, as the total chromium in water is composed of the sum of hexavalent and trivalent chromium species. The determination of total chromium involves a complete oxidation of the samples through potassium peroxodisulfate oxidation in sulfuric acid under 150 °C for an hour, as indicated by the supplier of the hexavalent chromium photometric determination kits (Macherey Nagel, Germany). The determination of hexavalent chromium of the oxidized sample following the indications of the supplier revealed the total chromium amount. 

Once analyzed the optimal operating conditions, these parameters were applied to treat the real wastewater from the urban WWTP which received industrial tannery effluents. 

### 2.5. Isotherm and Kinetic Data Analysis

Hexavalent chromium adsorption capacities were calculated and non-linearly adjusted to different kinetic and isotherm models which are commonly applied to batch adsorption treatments, following the equations indicated by Ojembarrena et al. [20]. The selected kinetic models were pseudo-first-order (PFO), pseudo-second-order (PSO), Elovich and intraparticle (IP) models. In the case of IP model, the adjustment must be divided into separated linear steps, as each linear step can be associated with the kinetic rate of the external or internal adsorption of chromium. The fittings of the different steps will be clearly shown by the consecutive number of the step. As an example, the first linear fitting of IP model to experimental data will be indicated as IP S1 and the second one, as IP S2. The chosen isotherm models to fit equilibrium data were Langmuir, Freundlich, Sips, Dubinin–Raduskevich (D-R) and Temkin models. The first three models offer information about the major mechanism of hexavalent chromium adsorption, while the other two show thermodynamic information of the adsorption process.

To obtain the preexponential factor, the energy of activation of the adsorption process and to reveal if the overall process is endothermic or exothermic, the Arrhenius equation (Equation (1)) was adjusted to the achieved values of PSO kinetic constants with temperature [29].

(1) $$\ln\left(k_{2}\right)=-\frac{E_{A}}{R\cdot T}+\ln\left(k_{0}\right)$$

Being *k*_2_ the values of the kinetic constants, *T*, the temperature (in K) corresponding to each kinetic constant, *R*, the constant of ideal gases (in J·K^−1^·mol^−1^)) and *k*_0_, the preexponential factor.

## 3. Results

### 3.1. CCNC Characterization

First, the process yield was determined to evaluate the cellulose losses during the process. Following the previously indicated procedure, a total process yield of 42.19% was calculated as a percentage of a solid mass of nanosized cellulose. This value is similar to the reported yield reached by Yang and van de Ven [21], who applied the same production process to a different raw material, such as softwood kraft pulp. Campano et al. [27] repeated the same process with a similar pulp yield of 54.9% as CCNC. The variation in the resulting yields can be associated with the higher or lower solubilization of cellulose when it is oxidized. According to Yang and van de Ven [21], the fraction of non-CCNC cellulose is kept as dissolved cationic cellulose.

The aldehyde content of the DAMC sample is a measurement of the yield of the dialdehyde formation reaction. In accordance with the indications of Campano et al. [27], the maximum amount of aldehyde groups if the dialdehyde formation reaction with NaIO_4_ yields a 100% is 9.24 mmol·g^−1^. In the case of the synthetized DAMC in the present study, the measured value was 1.83 mmol·g^−1^, which represents an aldehyde formation yield of 19.76%. This value is relatively low compared to the reaction conversion obtained by Campano et al. (3.84 mmol·g^−1^) [27] when starting with softwood pulp under the same conditions. These differences can be explained by the cellulosic source used as a raw material. In the case of the application of cotton linters, this material has a low percentage of reactive amorphous regions, since its crystallinity index (Cr·I) is high (72–87%) [30]. Oppositely, eucalyptus cellulose used as raw material for softwood pulp production shows lower Cr·I values (52–58%) [31], explaining the increased conversion yield obtained by Campano et al. On the other hand, the dialdehyde cellulose synthetized by Otoni, et al. [32] under severe temperature conditions (50 °C) only reached 0.6 mmol·g^−1^ of aldehyde conversion, due to the low periodate dosage (4.6-fold lower). Therefore, the obtained result of 1.83 mmol·g^−1^ can be considered an intermediate value compared to other bibliographic results. 

To evaluate the performance of the cationization reaction, the number of cationic groups was determined. The yield of the cationization reaction is obtained from the previously measured aldehyde content in DAMC. The result of the titration revealed a total of 1.02 mmol·g^−1^ of cationic groups in the produced CCNC. This value indicates a cationization reaction yield of 55.58%. This efficiency is much higher than the one obtained by Campano et al. (33.42%) [27], who obtained a similar final amount of cationic groups of 1.31 mmol·g^−1^. The differences in trends between the similar number of cationic groups of both materials while a high gap in reaction yield are explained by the previously determined number of aldehyde groups. Two cationized cellulose samples could show a similar number of cationic groups, like in this case. When one of them shows a much larger number of aldehyde groups, which would be the case with Campano et al., its yield of transformation of aldehyde groups into cationic groups would be much smaller than in the case of the present study, as most of the aldehyde groups in the cellulose sample would stay untouched. Another case of similar cationization yield was reached by Yang and van de Ven (60%) [21], whose total cationic group value was 1.68 mmol·g^−1^. In both cases, the initial raw material was softwood pulp, which can be the main cause of the apparent difference between measurements of cationic groups. In contrast, Otoni et al. [32] found a relatively low value of cationization yield and cationic groups (33% and 0.2 mmol·g^−1^), showing that the process followed by the rest of the studies, including the present one, was more efficient in terms of the formation of cationic groups on the cellulose surface.

The evaluation of CCNC surface anion attraction capacity was determined through anionic demand. The anionic demand of the synthetized CCNC material was 675.2 μeq·g^−1^. This value is close to other results achieved in cationization reactions of lignocellulosic nanomaterials. In the case of the lignin cationization with glycidyltrimethylammonium chloride synthesis performed by Wahlström et al. [33], 513 μeq·g^−1^ were achieved, and this material was efficiently applied to remove sulfate anions from wastewater, so the larger anionic demand found on CCNC would be a great value to attract and remove chromate anions from water.

The Cr·I was evaluated to define the amount of the amorphous cellulose region removed during the production and purification process through heat treatment. In this case, the achieved Cr·I was up to 81.87%. This value is higher than the Cr·I of the CCNC reported by Yang and van de Ven [21], which was 67%. The main reason for these varied results is explained by the source of lignocellulosic material. As explained before, the Cr·I values of the initial raw materials are critical. Cotton linters show a larger Cr·I than the softwood kraft pulp used by Yang and van de Ven. That high Cr·I cotton linters values are indicators of the obtention of much higher Cr·I values in the synthetized CCNC, as the synthesis process involves the removal of the amorphous zones from the cellulose chain.

The elemental analysis of the cellulose sample revealed an average composition of 38.89% of carbon, 6.19% of oxygen and 3.99% of nitrogen. The content of cellulose in cotton linters is above 90% [34]. Cellulose is a homopolymer composed of β(1–4) D-glucopyranose units with only carbon, oxygen and hydrogen atoms in its composition, according to Chen et al. [35]. This means that the presence of nitrogen can only be associated with the successful generation of the quaternary ammonium groups generated on the surface of the material. 

### 3.2. Hexavalent Chromium Adsorption Kinetics on CCNC

#### 3.2.1. Effect of pH

**Figure 2:**
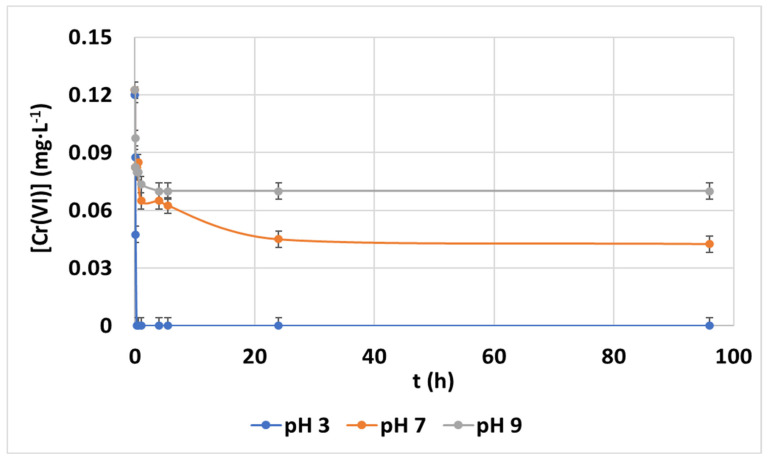
Evolution of hexavalent chromium concentration (mg·L^−1^) during adsorption batch experiments with CCNC with contact time at 0.1 mg·L^−1^ of initial chromium concentration, 100 mg·L^−1^ of CCNC dosage at room temperature at pH values 3 to 9.

The effect of pH (from 3 to 9) during the hexavalent chromium adsorption with CCNCs was analyzed as summarized in Figure 2. The experiments were performed at room temperature with an initial chromium concentration of 0.1 mg·L^−1^ and an adsorbent dosage of 100 mg·L^−1^. A complete abatement of hexavalent chromium is achieved at pH 3. Equilibrium is rapidly reached (15 min), with more than 60% of total removal in less than 5 min. At pH 7 and 9, the kinetics were also fast, but the removal yield found at equilibrium was lower (65.3% and 42.8%, respectively). The fact that the optimum pH is acidic is common in the bibliography, as monovalent chromate species (HCrO_4_)^−^ at pH conditions between 2 and 4 are easy to be attracted by the CCNC surface, and this species has an ion:active site adsorption ratio of 1:1. However, non-charged chromic acid H_2_CrO_4_ is mostly present when pH < 2, and it shows low adsorbate:adsorbent interaction and divalent chromate species (CrO_4_)^2−^ is predominant at pH > 6 and it requires an ion:active site adsorption ratio of 1:2, which is less efficient in terms of adsorbent usage. This optimal pH value for hexavalent chromium adsorption agrees with the previously obtained one by Ojembarrena et al., Qiu et al. and Peng et al. by applying hydrophobized CNFs, polyethyleneimine facilitated ethyl cellulose and polyvinylimidazole-modified cellulose, respectively [20,36,37].

**Figure 3:**
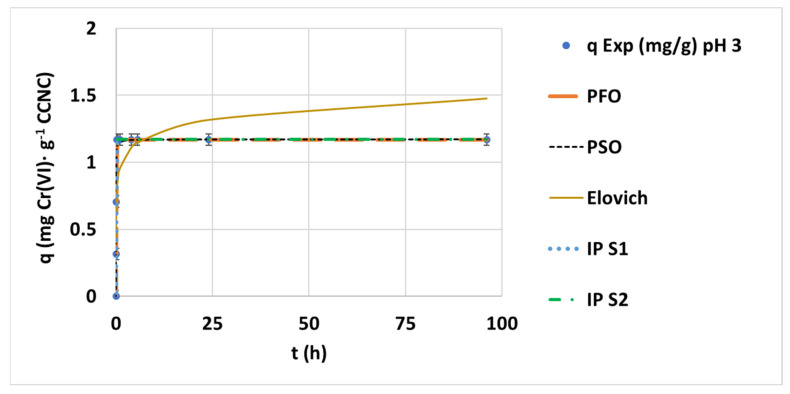
Evolution of the kinetic adsorption capacities at 0.1 mg·L^−1^ of initial chromium concentration, 100 mg·L^−1^ of CCNC dosage and room temperature under pH 3 conditions and the kinetic fitting of the PFO, PSO, Elovich and IP models.

The experimental hexavalent chromium concentration data were converted into adsorption capacity values to allow the kinetic adjustment to different models. The resulting fitted curves for adsorption capacity values at pH 3 conditions are presented in Figure 3.

**Figure 4:**
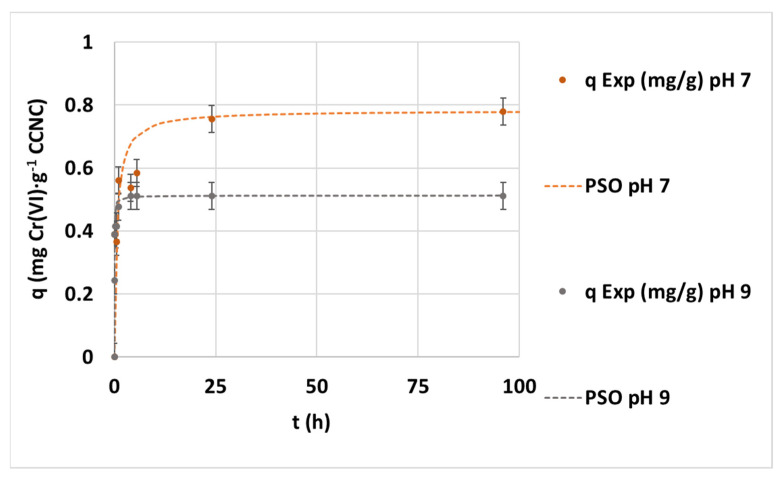
Evolution of the kinetic adsorption experiment at 0.1 mg·L^−1^ of initial chromium concentration, 100 mg·L^−1^ of CCNC dosage and room temperature under pH 3, 7 and 9 conditions and the kinetic fitting of PSO.

The adjustment of the different curves reveals that PFO (R^2^ = 0.9956) and PSO (R^2^ = 0.9505) would represent the saturation curve better than the Elovich model. The obtained kinetic parameters in the different pH experiments can be seen in Appendix A, Table A1. The IP study indicates the following clear two-step adsorption equation: a first rapid adsorption and a second flat straight line during saturation. The plot of the PSO kinetic model fitting to each tested pH condition data can be seen in Figure 4. This figure explains the clear trend of adsorption capacity reduction while increasing pH from 7 to 9. The PSO equation is an accurate representation of the experimental data at each pH. There is a strong minimization of adsorption capacities and rates with a pH increase. It can be confirmed that the pH rise involves a change from a two-step process to a multi-step adsorption. This effect is associated with mass-transfer limitations, the need for more active sites per adsorbed anion and the presence of competitive anions at alkali conditions, such as hydroxide or carbonate.

The results reveal that the most adequate pH between the tested ones to obtain high hexavalent chromium removal through CCNC is pH 3.

#### 3.2.2. Effect of CCNC Dosage

**Figure 5:**
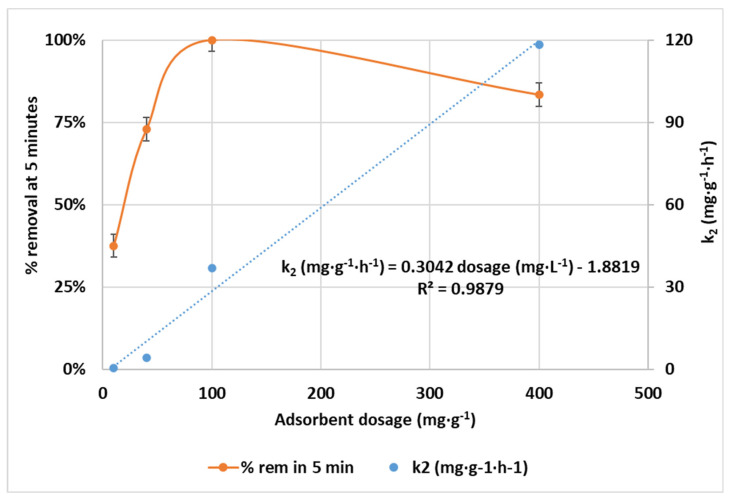
Evolution of removal of hexavalent chromium in 5 min of contact time (%) and PSO kinetic constants during adsorption batch experiments with CCNC at 0.1 mg·L^−1^ of initial chromium concentration, pH 3 and room temperature while varying CCNC dosage from 10 to 400 mg·L^−1^.

The adsorbent dosage was studied in the interval from 10 to 400 mg CCNC·L^−1^. Experiments were carried out with a contaminant concentration of 0.1 mg·L^−1^ at pH 3 at room temperature. The removal efficiency at short contact times (5 min) and the kinetic constant for the different CCNC dosages are shown in Figure 5. Results indicate a complete depletion of chromium for a dosage of 100 mg·L^−1^ in just 5 min of contact and a linear correlation between dosage and the PSO kinetic constant. This dosage shows a great performance in terms of adsorption rate. On the other hand, possible interactions between CCNC particles were observed at 400 mg·L^−1^, as at initial contact times, the hexavalent chromium removal is lower than at 100 mg·L^−1^. This interaction could interfere with the chromium adsorption efficiency due to the formation of flocs or larger crystals [38]. Another option could be that the large concentration of cationic charges caused a constant repulsion between CCNCs and affected the adsorption capacity and rate. It is also remarkable that much lower dosages, such as 40 mg·L^−1^ would allow the full adsorption of hexavalent chromium in a relatively short contact time of 60 min. Nevertheless, the increased kinetics revealed by the kinetic essay with 100 mg·L^−1^ suggests that this is the real minimum dosage value to obtain adequate adsorption rates, as there is a 12-fold reduction in the necessary time for total removal with just a 2.5-fold increase in dosage. This value will be established as the best operating dosage and will be applied in the rest of the experiments. The obtained values are relatively lower than those mentioned in the analysis of several studies performed by Aigbe et al. [38], which are dedicated to the application of nanomaterials, such as carbon, polymer and metallic nanomaterials, in hexavalent chromium adsorption. These authors showed that there is a strong correlation between adsorbent dosage and pollutant removal yield, being 500 mg·L^−1^ the established the lowest dosage for a minimum removal of 95% and diminishing to 70% when the dosage was below 200 mg·L^−1^. 

The obtained optimal nanocellulosic adsorbent dosage of 100 mg·L^−1^ is several times lower compared to previous studies in the field. For example, Yang et al. needed 400 mg·L^−1^ of BC/poly(m-phenylenediamine) nanoparticles for an efficient removal of hexavalent chromium, due to the large chromium concentration treated in this study (200 mg·L^−1^) [18]. As a comparison, while treating similar concentrations, in our previous study using hydrophobized CNFs, a dosage of 500 mg·L^−1^ was needed to treat concentrations below 1 mg·L^−1^ [20]. Peng et al. developed a polyvinylimidazole-modified cellulose adsorbent whose tested dosage was up to 1000 mg·L^−1^ [37]. The largest analyzed dosage between these studies was obtained by Zeng et al., who added 30,000 mg·L^−1^, two orders of magnitude higher than most of the studies. The main differences between the analyzed studies can be associated with the different concentrations and also with the synergistic effect of cationization performed in this study, which involves more attraction of chromate anions, being a more efficient adsorbent than other tested modified cellulosic materials. This aspect explains the low dosage of CCNC to obtain fast and efficient removal of this contaminant. 

Chromium adsorption capacities were calculated and adjusted to different kinetic models to evaluate the best representation of the experimental results while varying the CCNC dosage. The calculated kinetic parameters are shown in Appendix B, Table A2. The trend indicated in Table A2 is similar to the pH effect, where PFO (R^2^ = 0.9967) and PFO (R^2^ = 0.9907) kinetics showed a well-fitting of the experimental data compared to the case of the Elovich model, and the approximation of the PFO kinetic model to the growth step and the saturation line of the curve is represented with accuracy. The reduced amount of active sited due to the addition of low adsorbent dosage present in the 40 mg·L^−1^ causes the need for three IP steps. Even happening a fast reduction of hexavalent chromium to trivalent species, such a minimal number of active sites forces the accumulation of a much larger amount of chromate anions in the boundary layer of the nanocrystals. In any case, only half-hour of close contact allowed the removal of more than 90% of the total hexavalent chromium from the solution while achieving a relatively high adsorption capacity (2.80 mg·g^−1^) when treating low-concentration solutions. This equilibrium capacity value depicts a five-fold increase when compared to other nanocellulosic materials in the same order of magnitude, such as hydrophobized CNFs [20]. 

#### 3.2.3. Effect of Initial Chromium Concentration

To evaluate the limits of this process, the efficiency of the CCNC adsorbent in the removal of hexavalent chromium solutions at higher concentrations has been tested. The equilibrium data reached from these experiments were necessary to perform the isotherm analysis. These experiments were carried out under variable initial hexavalent chromium concentrations from 0.1 to 70 mg·L^−1^. The different kinetic constants reached during the fitting of modifying initial hexavalent chromium concentration data are explained in detail in Appendix C, Table A3 and Table A4. The previously selected operating conditions of pH 3, 100 mg·L^−1^ adsorbent dosage and room temperature were applied.

**Figure 6:**
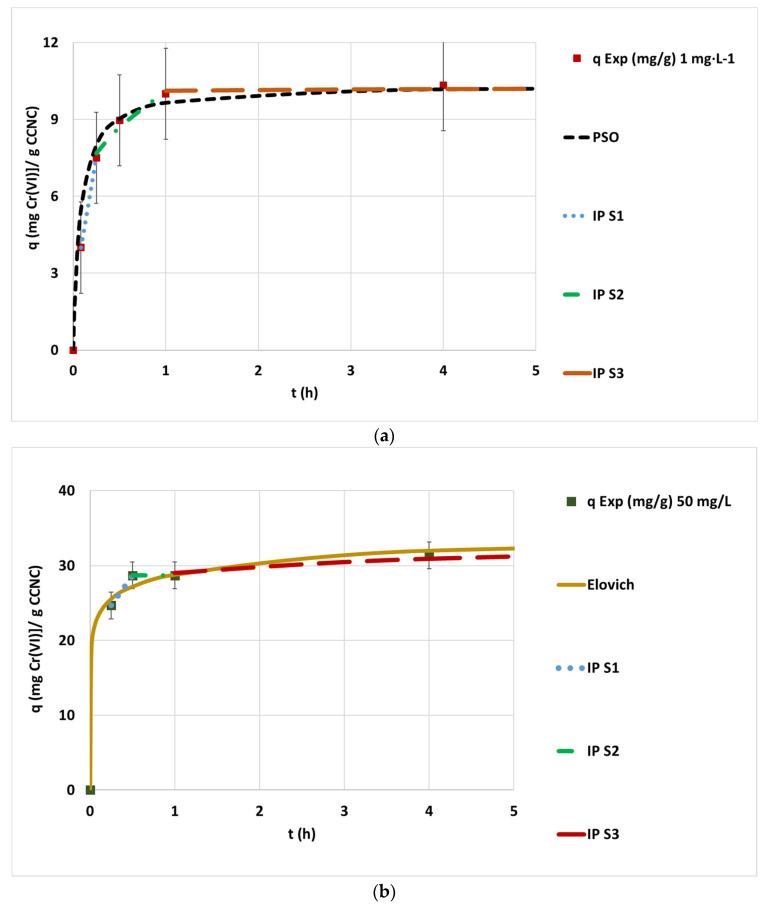
(**a**) Evolution of the kinetic adsorption experiment at 1 mg·L^−1^ of initial chromium concentration under pH 3, room temperature and 100 mg·L^−1^ of CCNC dosage and 5 h of contact time and the kinetic fitting of the PSO and IP models; (**b**) 50 mg·L^−1^ of initial concentrations under the same experimental conditions and the kinetic fitting of the Elovich and IP models.

The obtained curves showed an opposite trend between the low chromium concentration experiments (0.1–1 mg·L^−1^) and the highly concentrated tests (>5 mg·L^−1^). This tendency is shown in Figure 6a,b, where the experimental data achieved at 1 mg·L^−1^ and 50 mg·L^−1^ and the corresponding optimized kinetic adjustments are plotted. 

In the case of low-concentration experiments, the chromium was totally adsorbed by the applied dose of CCNC. For this reason, these curves show a common saturation kinetic shape with an initial growth step and a late plain line at the end corresponding to the final equilibrium value. The kinetic behavior changed when the concentration was over 5 mg·L^−1^ because the number of chromate anions may have overpassed the number of accessible active sites. For this reason, the complete depletion of hexavalent chromium present in water was not found after that tested concentration. The shape of the obtained curves when treating hexavalent chromium-concentrated water reveals the presence of mesas or plain intermediate steps after the initial growth phase. Both the presence of intermediate plateau steps, which are typical indicators of multilayer adsorption, and the occurrence of different mechanisms between treating high- and low-concentrated wastewaters and this fact were also indicated by Ojembarrena et al. and Pourfadakari et al. when adsorbing hexavalent chromium onto different lignocellulosic materials [20,39]. The adsorption mechanism might be explained by this sequence: the first stage before the plateau could be dominated by the same mechanism of process in a sequence of adsorbate-adsorbent interaction and hexavalent chromium reduction to trivalent chromium. Once equilibrium was reached, a strong rate minimization was likely to happen due to the saturation of active sites and lowered catalytic activity caused by two main factors. The first factor is the accumulation of the formed trivalent chromium onto the CCNC active sites, which must leave the active site toward the bulk through the micro and macropores or get attached to other active groups of cellulose far from the cationic groups. The second factor is due to the difference between cationic groups and reducing groups. While the number of cationic groups attracting anions to the CCNC surface that are simultaneously reduced would be relatively constant due to the sequential rapid adsorption of hexavalent chromium and desorption of generated trivalent chromium, which frees the active site continuously, the quantity of reducing groups that have been involved in the reduction reactions would be constantly going down as these groups may not be regenerated once they are spent. These facts would cause a severe lowering of the adsorption rate, which can be seen in the plateau. The second growth step would happen when part of the trivalent chromium leaves the occupied active sites and the hexavalent chromium present in the micropores could finally get into the active sites and be converted into cationic trivalent groups during a slow process. Then, the extremely high concentration of hexavalent chromium in the bulk and the presence of a positive charge of brand-new trivalent chromium cations close to the active sites might generate enough driving force to push chromate anions from the bulk to the boundary layer and then to the macro and micropores of the particles to fill the recently freed active sites, but this adsorption step would be much slower than the initial growth step. 

These results can be confirmed by the comparison between the curves and the adjustments of the low-chromium-concentrated adsorption test of 1 mg·L^−1^ and the highly concentrated experiment of 50 mg·L^−1^ (Figure 6a,b). The first adsorption data of 1 mg·L^−1^ (Figure 6a) seems to approach PSO kinetics, with high correlation parameter values (R^2^ = 0.9886), and a three-step IP mechanism ruled by two growing straight lines with decreasing slope and a last saturation straight line. As the linear regression of the first IP step has an intercept close to the origin, the rate-controlling step can be considered IP diffusion [40]. The other adsorption experiment of 50 mg·L^−1^ (Figure 6b) showed a low correlation coefficient to PFO and PSO saturation models, a three-step IP model with a first fast adsorption process (15–30 min), followed by a plateau (30–60 min) and a second slow adsorption process (from 1 h to equilibrium). The IP model adjustment of the first step revealed an intercept far from the origin, meaning that external diffusion is the rate-controlling step of the process while operating under high-concentration conditions [40]. This fact indicates a mechanism of modification between the low- and high-concentrated wastewaters. The experimental data seen in Figure 6b was well-fitted to the Elovich model. This fact happened to each experiment depicted as highly concentrated (from 5 to 70 mg·L^−1^), suggesting a common mechanism associated to all of them. This model is typically associated with the chemisorption processes of gases on solid surfaces with a low or no desorption process, where the adsorption rate decreases while the surface coverage rises up [41]. This model has been effectively applied with the adjustment of batch and ion exchange adsorption experimental results of several metals from water onto different materials, such as Cr(III) and Cu(II) on peanut shell, Cr(VI), Pb(II) and Zn(II) on rice husk ash or Zn(II), Fe(III), Cu(II), Co(II) and Ni(II) on solvent-impregnated resins [42,43,44]. The results mentioned by these authors were achieved while treating concentrated solutions of metals between 10 mg·L^−1^ and several g·L^−1^, confirming that the observed behavior is only representative of highly chromium-polluted wastewaters.

#### 3.2.4. Effect of Temperature

**Figure 7:**
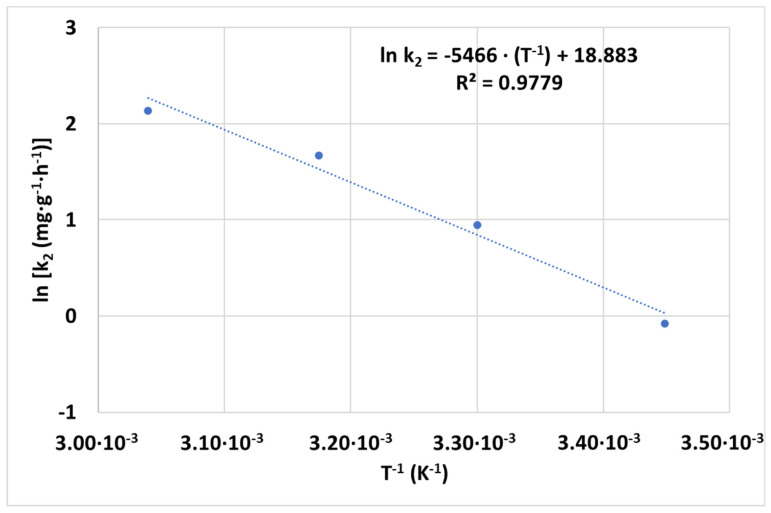
Evolution of ln(k_2_) with T^−1^ (K^−1^) and linear fitting of Arrhenius equation.

The effect of rising temperatures on hexavalent chromium removal through CCNC was analyzed. Other operation parameters that were optimized before were kept constant, including pH 3, the adsorbent dosage of 100 mg·L^−1^ and the initial concentration of 1 mg·L^−1^. The experimental data were adjusted to PSO kinetics reaching high correlation parameter values. The obtained kinetic constants at each temperature were then adjusted to the Arrhenius equation (Equation (1)), whose plotted results can be seen in Figure 7. The PSO kinetic parameters obtained under each tested temperature and Arrhenius’ energy of activation and preexponential constant can be seen in Appendix D, Table A5.

The trend seen in Figure 7 depicts an accurate fitting of PSO kinetics to the varying temperature experimental data (R^2^ > 0.98 achieved in each adjustment) and a clear increase in the kinetic constant while the temperature is rising. Each curve corresponds to an increment of 12–14 °C and involves a two-fold average increase in the k_2_ value between experiments. The last slope was almost vertical, meaning an instant removal of hexavalent chromium. 

Adsorption of hexavalent chromium coupled to reduction can be explained by an equilibrium process. Once a hexavalent chromium anion is adsorbed, this anion can follow the reverse path to get desorbed or it can be converted into trivalent chromium in the active site. Logically, the direct adsorption process has an enthalpy sign, while the reverse desorption follows the equivalent enthalpy value with the contrary sign, as explained by Hess’ law. The trend of the adsorption rate with temperature would show the enthalpy sign. The temperature rise would deal with the following two possibilities: a faster hexavalent chromium adsorption in the case of an endothermic direct process; or a slower or null adsorption in the case of an exothermic direct process, as in this case, the desorption process is favored by the temperature increase and its rate would be greater than the adsorption rate. The enhancement of the process kinetics while increasing temperature demonstrated that the overall process of adsorption of hexavalent chromium onto CCNC is an endothermic process (ΔH^0^ > 0). Another relevant aspect is the value of the energy of activation, E_A_ = 45.45 kJ·mol^−1^. This value is relatively higher but in the order of magnitude of the energy of activation found by applying V, Ti-bearing magnetite (VTM) humic acid-modified particles and Magnetite/3D-Printed Wollastonite Hybrid, where 17.408 and 14.49 kJ·mol^−1^ were calculated, respectively [45,46]. The difference in Temkin’s heat of sorption can be caused by the sequential adsorption-reduction process involved in the CCNC application instead of pure adsorption processes. Nollet et al. indicated that the barrier between physical and chemical processes can be established if a process involves a total activation energy below or over 40 kJ·mol^−1^ [47], meaning that the overall removal process through adsorption and reduction of hexavalent chromium would be energetically considered a process between chemical and physical processes. 

### 3.3. Hexavalent Chromium Isotherm Study

**Figure 8:**
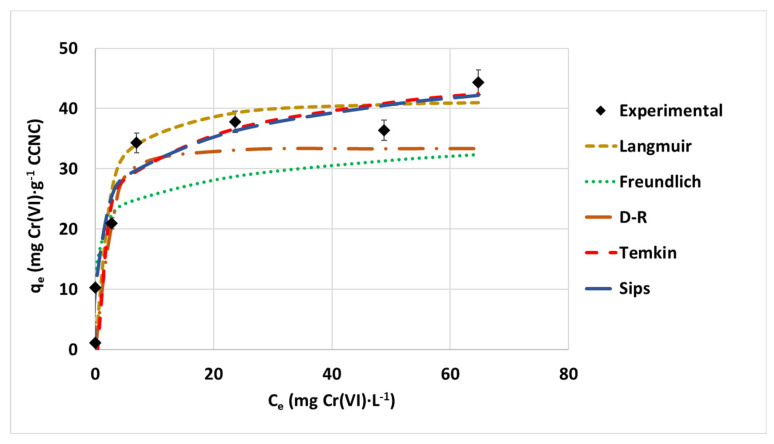
Isotherm experimental data of hexavalent chromium on CCNC and isotherm model adjustment of the Langmuir, Freundlich, Temkin, D-R and Sips equations.

The isotherm study was performed by adjusting the maximum adsorption capacity values at different initial concentrations for the selected isotherm models. The representation of these equilibrium data would be necessary to understand the adsorption equilibrium mechanism. The plotted experimental data and the adjustment of the different isotherm model equations are seen in Figure 8. The evolution of this data suggests a favorable to a strongly favorable mechanism of hexavalent chromium adsorption onto the CCNC surface, according to the classification shown by Kaushal and Singh [48]. The curve shows an intermediate trend between the Langmuir (hyperbolic shape, saturation mechanism, monolayer adsorption and homogeneous dispersion of energy and active sites over the adsorbent surface) and Freundlich models (exponential growth, multi-layer adsorption and dispersion of energy and active sites). The exponential growth shape of the initial part of the curve is combined with a second step with a lower slope at higher concentrations. For this reason, the non-linearly optimized parameters of Sips’ model, a mixed exponential-hyperbolic equation model, have the best correlation parameter adjustment (R^2^ = 0.9787), while both the Langmuir and Freundlich models show a lower and similar value of correlation parameter of R^2^ = 0.96. The resulting fittings are shown in Figure 8, and the adjustment parameters can be seen in Table 1.

The evaluation of the isotherm parameters reveals a favored interaction between hexavalent chromium and the CCNC surface. The value of the n_F_ parameter obtained from the Freundlich model is considerably higher than 1 (n_F_ = 8.4674). Other bibliographic values of n_F_ > 1, showing favorable hexavalent chromium adsorption, were achieved by the application of other kinds of nanocellulosic materials, such as polypyrrole surface-modified CNC [49,50], carboxylated CNC-polyethylenimine composite [51] or black wattle tannin-immobilized nanocellulose [52]. As well, the Langmuir’s saturation factor has a value of R_L_ < 1 in all the studied intervals of concentrations, showing an energetically favored adsorption [49]. These values have been previously mentioned for other cellulosic nanomaterials, such as hydrophobized CNF [20] and polypyrrole surface-modified CNC [49]. The R_L_ values obtained in the initial steps of adsorption are closer to 1, indicating a strong bonding interaction between adsorbent-adsorbate, which is reduced together with the R_L_ value along the adsorption process [53]. 

D-R and Temkin models were selected to fit experimental data and obtain relevant thermodynamic information about the process. These calculated or adjusted data can be seen in Table 1. The mean free energy of the process is established by D-R equations, reaching a final value of 0.748 kJ·mol^−1^. As this process was completely spontaneous, this thermodynamic parameter must be established as ΔG = −0.748 kJ·mol^−1^. This value is associated with a physical sorption of hexavalent chromium onto the surface (<8 kJ·mol^−1^) and is close to other reported values on fluorescent nanocellulose-based hydrogel incorporating titanate nanofibers (−0.724 kJ·mol^−1^) [54] and hydrophobized CNF (−2.24 kJ·mol^−1^) [20], with this value being slightly higher due to the mass transfer limitations of chromate anions through the CNF-coating agent. Other authors indicate a much higher value of ΔG of hexavalent chromium of (2,3-epoxypropyl) trimethylammonium chloride (EPTMAC)-modified CNC that is close to the barrier between physical and chemical processes (−7.84 kJ·mol^−1^) [55]. The main reason is caused by the acid hydrolysis-anionic CNC used as the base for surface modification. Even when EPTMAC is a cationization agent, if part of the CNC surface is not attacked by the cationization reagent, this would keep anionic groups unchanged, causing mass transfer limitations of chromate from the bulk to the active site, which are not present in CCNC.

The parameter b_T_ from Temkin is associated with the heat of sorption of the adsorption process, and the obtained value is 0.412 kJ·mol^−1^. This heat of sorption is slightly higher but in the same order of magnitude as other values reported in the bibliography for fluorescent nanocellulose-based hydrogel incorporating titanate nanofibers (0.129 kJ·mol^−1^) [54], hydrophobized CNF (0.19 kJ·mol^−1^) [20], and nanocellulose-based hydrogel incorporating silver nanoclusters (0.135 kJ·mol^−1^) [56]. The difference between these values can be due to the fact that the CCNC removal process covers a simultaneous adsorption-reduction process, which involves a higher exchange of energy. According to the criteria established by Choudhary and Paul [57], a value of b_T_ < 8 kJ·mol^−1^ is associated with low energetic interactions between adsorbent and adsorbate, typical of physisorption processes. 

### 3.4. Determination of Trivalent Chromium in Treated Samples

**Figure 9:**
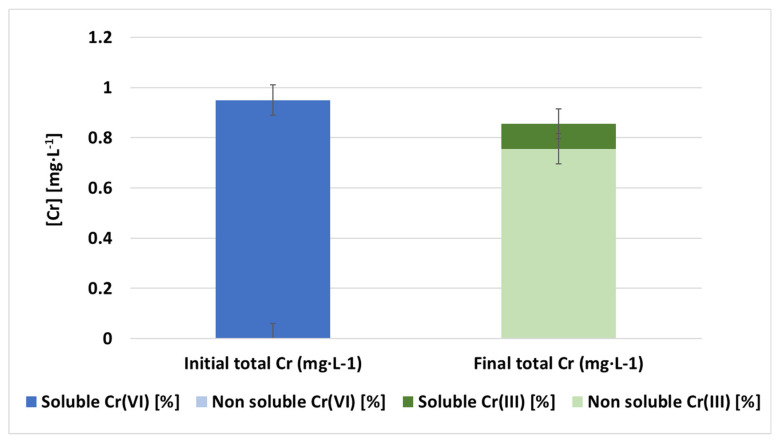
Initial and final concentrations of hexavalent and trivalent chromium in soluble and non-soluble fractions of treated water during adsorption batch experiments with CCNC for 24 h under pH 3, 100 mg·L^−1^ of adsorbent dosage and 1 mg·L^−1^ of initial hexavalent chromium concentration.

The determination of trivalent chromium was performed in treated samples with 1 mg·L^−1^ an initial hexavalent chromium concentration. The results of the initial and final concentrations of the soluble and non-soluble fractions of both hexavalent and trivalent chromium species are indicated in Figure 9. The soluble concentrations of hexavalent and trivalent chromium were measured in water samples, which were filtered with 0.45-micron syringe filters. The total concentrations of both species were determined by measuring them in water samples without any kind of filtration. Non-soluble concentrations were calculated by subtracting the total minus the soluble contents of each species. This hexavalent chromium solution was treated for 24 h under pH 3, 100 mg·L^−1^ and room temperature conditions.

**Figure 10:**
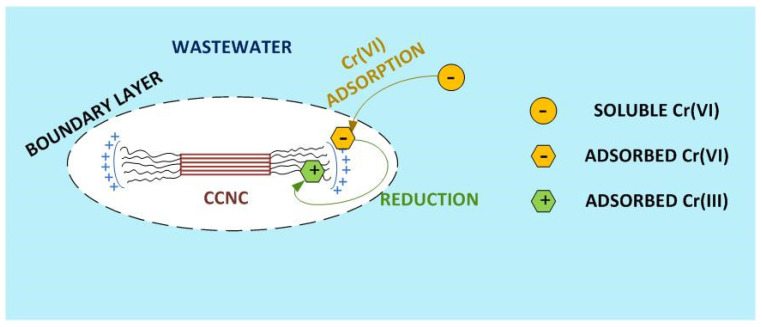
Graphical scheme of the adsorption-reduction mechanism of hexavalent chromium through CCNC.

The results shown in Figure 9 depict that a complete depletion of hexavalent chromium is reached in both soluble and non-soluble (adsorbed on CCNC) fractions, meaning that CCNC could efficiently reduce all the present hexavalent chromium into the less toxic trivalent species. In addition, the distribution of chromium in the water-adsorbent matrix changes completely. Obviously, the initial hexavalent chromium was completely dissolved. After the treatment with CCNC, hexavalent chromium was totally removed and most of the present chromium (89%) is found as trivalent chromium in the non-soluble fraction. This happens due to the instant reduction of hexavalent chromium to a trivalent species and its simultaneous fixation onto the surface of the CCNC. According to the Pourbaix diagram of chromium [58], Cr^3+^ is the predominant species at pH 3 in a wide interval of reduction-oxidation potential values from −250 mV to 800 mV. This fact suggests that this is the main present chromium species after CCNC addition to hexavalent chromium-polluted water. According to previous articles in the field of lignocellulosic adsorbents on trivalent chromium removal, cellulose hydroxyl groups would play a major role in the fixation of trivalent chromium to the adsorbent surface [59,60]. Only 11% of the generated trivalent chromium moved from the active sites back to the bulk and its final soluble concentration in water was relatively low, averaging 0.10 mg·L^−1^. The suggested hexavalent chromium adsorption-reduction removal mechanism is explained in Figure 10.

This fact would indicate a successful removal of the chromium from the water bulk. According to the national limits of discharge compiled by Vaiopoulou and Gikas [61], the final soluble total and hexavalent chromium values comply with almost all the established national discharge limits for the aquatic environment in the European Union and these values are lower than all the established national discharge limits for specific industrial sectors in the European Union, which include metal finishing, pigments and tanning.

### 3.5. Application of CCNC to Urban Wastewater with Tannery Effluents

To evaluate the performance of CCNC as a hexavalent chromium adsorbent in real conditions, this material was applied to the treatment of a real wastewater. Samples were taken from an effluent of a WWTP that receives the industrial effluents from tannery industries, which contain hexavalent chromium contamination. Adsorption treatment was focused on the removal of hexavalent chromium and compliance with its local limit of discharge (0.1 mg·L^−1^). The physical-chemical characterization of the treated wastewater is shown in Table 2.

**Figure 11:**
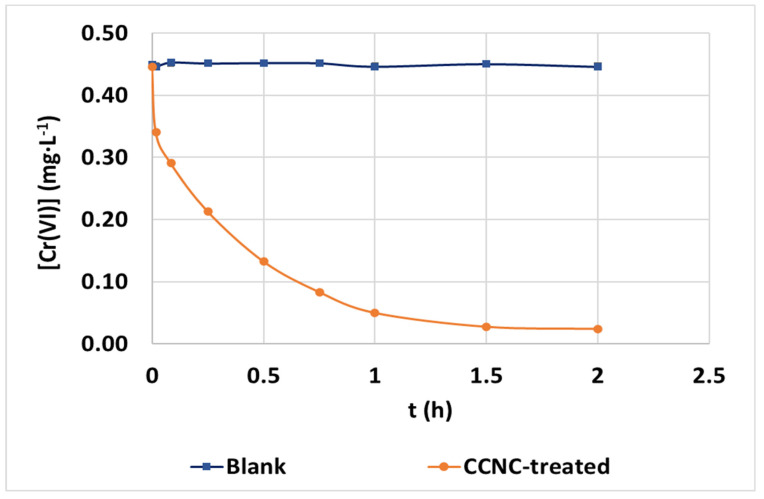
Evolution of hexavalent chromium concentration (mg·L^−1^) in blank and treated samples during adsorption batch treatment with CCNC of real wastewater under pH 3 and 100 mg·L^−1^ of adsorbent dosage.

To perform this experiment, the previously optimized conditions of pH (pH 3) and adsorbent dosage (100 mg·L^−1^) were applied. The resulting evolution of hexavalent chromium treated by CCNC adsorption with this complex matrix can be found in Figure 11. The kinetic parameters and constants reached while fitting real wastewater treatment data can be found in Appendix E, Table A6. Observing the obtained results and considering the high concentration of dissolved salts seen in the electrical conductivity result, there is still a strong attraction between hexavalent chromium and CCNC. Equilibrium concentrations were close to the total abatement of chromium (0.02 mg·L^−1^) and only 40 min of contact time was necessary to reach compliance with legal restrictions. In this case, the kinetics were slower than in the case of synthetic solutions due to the presence of interferent species present in real wastewater, such as sulphate anions. The best fitting of experimental data was achieved by both PFO (R^2^ = 0.9920) and PSO (R^2^ = 0.9897), in the same way as treating low-concentrated synthetic solutions. The IP model shows a three-step process. The first long step indicates that internal diffusion is the rate-controlling step, as the linear regression intercepts next to the origin. 

**Figure 12:**
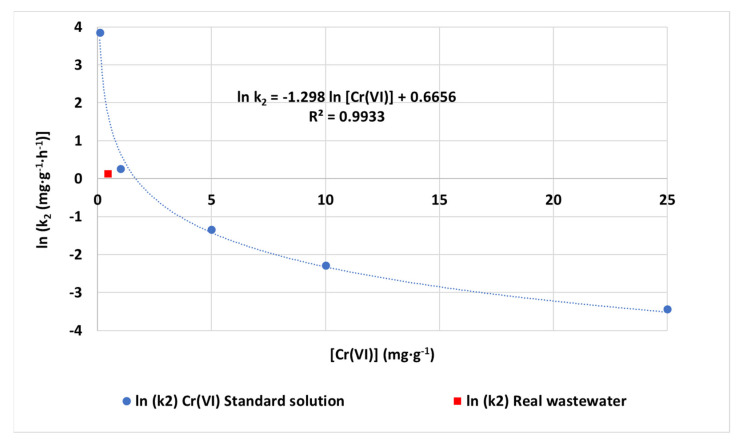
Evolution of PSO kinetic constant (k_2_) while treating synthetic waters under varied initial chromium concentrations (0.1–25 mg·L^−1^) and real wastewater at pH 3 and 100 mg·L^−1^ of adsorbent dosage and logarithmic fitting of k_2_ values of synthetic waters.

The evaluation of the adjustment accuracy of the achieved kinetic parameters from synthetic solution to real wastewater was carried out. The result of the logarithmic fitting of PSO kinetic constants under varied low chromium concentrations and the obtained PSO kinetic constant when treating real wastewater can be seen in Figure 12. According to the initial concentration (0.45 mg·L^−1^), if the result was obtained from synthetic water, the expected k_2_ kinetic constant would be estimated through logarithmic estimation as k_2,est_ = 5.49 mg·g^−1^·h^−1^ between those obtained for 0.1 mg·L^−1^ (47.28 mg·g^−1^·h^−1^) and 1 mg·L^−1^ (1.31 mg·g^−1^·h^−1^). The final value of PSO is slightly reduced compared to this value but close to the prediction of the logarithmic adjustment. The inhibition caused by the presence of other ions and sorbates reduced its value to k_2,exp_ = 1.14 mg·g^−1^·h^−1^. This fact indicates that kinetic experiments with synthetic waters under controlled conditions can be used to predict the order of magnitude of kinetic constants while treating contaminated real wastewaters with good accuracy in the order of magnitude. 

Faster kinetics would be feasible by adding higher CCNC dosage, as the applied dosage was tested under favorable conditions (synthetic water with extremely low electrical conductivity), but the achievement of high removal rates and yields in polluted wastewater through these selected conditions is remarkable. This experiment confirms the specificity of CCNC with hexavalent chromium when large concentrations of interferent species are present and the applicability of this material in the treatment and legal compliance of real effluents suffering from hexavalent chromium contamination.

## 4. Discussion

The hexavalent chromium results achieved by CCNC were contrasted with previous bibliographic results of this metal’s adsorption with different lignocellulosic materials, nanomaterials and other adsorbents. The comparison between the performance of these mentioned materials with CCNC is detailed in Table 3.

Compared to the compiled data from the bibliography, the operation of the synthetized CCNC shows two highlights when compared to other materials: the fast approach to equilibrium, which is commonly achieved in 5 min, especially when treating low-concentrated solutions, and the low necessary dosage to reach 100% of removal. The only material able to reach more than 90% of removal in just 10 min under certain circumstances was carboxymethyl NC-stabilized zerovalent iron nanoparticles synthetized by Kumar et al. [62], but the dosage requirements are three times higher than CCNC and require the presence of nZVI nanoparticles to obtain an accurate reduction of hexavalent chromium, which in the case of the present material is reached by the modified-cellulose itself. In terms of adsorbent dosage, adsorption through humic acid-Fe(II) system structured on V, Ti-bearing magnetite surface also required 100 mg·L^−1^ of total dosage but low adsorption capacity (3.67 mg·g^−1^) and extended contact times were required (700 min) [45]. This fact suggests that the further implementation of the material would involve an adsorbent dosage several times higher than the optimal found in this study. This factor is so critical that CCNC is the only material that reached the complete removal of hexavalent chromium with an extremely reduced dosage of 40 mg·L^−1^. This dosage is so minimal that in most of the presented papers it is not even considered in the dosage optimization, but its application with CCNC was carried out with success in 1 h of treatment. 

The obtained adsorption capacity of CCNC (44.36 mg·g^−1^) is in the order of magnitude of most of the analyzed adsorbents. This material clearly overpassed the experimental results under lower concentrations of rice husk powder (1.4 mg·g^−1^) [43] and humic acid-Fe(II) system structured on V, Ti-bearing magnetite surface (3.67 mg·g^−1^) [45]. Lower values were found by applying EPTAC-modified CNC (22.99 mg·g^−1^) [55] and lignocellulosic substrate from wheat bran (35 mg·g^−1^) [63] under comparable conditions for the maximum tested hexavalent chromium concentration (dozens of mg·L^−1^), due to the probable presence of certain anionic repulsion in the first case and the mentioned low reduction capacity of the second material. The following other materials tested under similar conditions reached slightly higher adsorption capacities: hydrophobized CNF (70.38 mg·g^−1^) [20], carboxymethyl NC-stabilized nZVI (87.71 mg·g^−1^) [62] and activated carbon synthetized from *Z. jujuba* (60 mg·g^−1^) [64]. This last material does not show a significant difference, while the variation in the values compared with CNF hydrogel can be associated with the non-favored shape of the curve, meaning that chromium adsorption was only favored when large concentrations were applied. In the case of carboxymethyl NC-stabilized nZVI, it can be associated with the well-known reduction activity of nZVI present in the adsorbents. 

In general terms, CCNC adsorbent material shows an adequate adsorption capacity with an extremely reduced adsorbent consumption needed in remarkably short adsorption contact times. 

## 5. Conclusions

The application of CCNC to hexavalent chromium adsorption was efficiently performed, reaching the complete removal of the contaminant in only 5 min under the selected operating conditions. Furthermore, all the soluble hexavalent chromium present in water samples was converted into trivalent chromium specie, mainly attached to the adsorbent (89% of the total), which represents a significant advantage with respect to the state of the art, as the application of this material avoids the harmful effects of hexavalent chromium since trivalent chromium is less toxic than hexavalent chromium.

This nanomaterial was synthetized from lignocellulosic raw materials, and the cationization reaction was carried out successfully, reaching a high degree of crystallinity (Cr·I = 81.7%), cationic groups (1.02 mmol·g^−1^) and anionic demand (675.2 μeq·g^−1^) levels, which are great indicators of the material adequation for the adsorption purpose. The operation parameters were chosen to obtain the best conditions and results, being pH 3 and an adsorbent dosage of 100 mg·L^−1^. The complete abatement of chromium was reached with 40 mg·L^−1^, which is a low dosage compared to the bibliography values. These conditions allow the total depletion of hexavalent chromium in water samples up to 1 mg·L^−1^. The dominant kinetic mechanisms in the adsorption of low concentrated solutions up to 1 mg·L^−1^ are PFO and PSO kinetics, which could be interpreted as a saturation kinetic curve that follows an exponential growth. The IP diffusion model in this situation revealed a continuous growth in two or three steps under a predominant internal diffusion rate. On the other hand, highly chromium-polluted waters above 5 mg·L^−1^ of concentration followed an Elovich kinetic curve, and the IP diffusion model depicted a sequence of fast rate-plateau-slow rate, where external diffusion was the dominant step. The results of the isotherm study showed a great fit to the Sips model and favorable adsorption, meaning an adsorption mechanism with intermediate behavior between the multilayer and heterogeneous distribution of active sites seen on the Freundlich model and the monolayer and smooth distribution of surface energy and active groups explained by Langmuir. The maximum experimental capacity was 44.36 mg·g^−1^ at 70 mg·L^−1^. The thermodynamical analysis performed through the calculation of the mean free energy of adsorption (−0.748 kJ·mol^−1^) and heat of sorption (0.412 kJ·mol^−1^) values from the D-R and Temkin isotherms are indicators of physical sorption but higher than the bibliographical values reached for hexavalent chromium adsorption. According to the rising-temperature experiment indicated an endothermic trend in the overall adsorption process. Together with the high activation energy (45.45 kJ·mol^−1^, high for a sorption process and between the limits of physical and chemical processes (40 kJ·mol^−1^)), and the relatively high-energy involving process revealed by the Arrhenius equation, it was suggested that the presence of other sequential steps combined with an adsorption mechanism involving a chemical reaction must be considered. The presence of only trivalent chromium in treated samples as total chromium, mainly attached to CCNC (89% of the total), confirmed the hypothesis. The final application of the material under the best-operating conditions to real wastewater heavily contaminated with chromium and dissolved salts was feasible, reaching fast kinetics even when other pollutants were present, removing the entire content of hexavalent chromium and allowing the legal compliance of the discharge limit in 40 min of treatment. These results highlight the high potential of CCNC for adsorption processes thanks to the fast kinetics, the low dosage needed and the good adsorption performance achieved.

## Figures and Tables

**Table 1:** Adjusted isotherm model parameters to hexavalent chromium adsorption onto CCNC experimental data.

Model	Parameters	Values
Langmuir	Isotherm parameters	k_L_ (L·mg^−1^) = 0.6103 q_e,L_ (mg·g^−1^) = 42.02 R_L_ (C_0_ = 0.1 mg·L^−1^) (-) = 2.29·10^−2^ R_L_ (C_0_ = 70 mg·L^−1^) (-) = 0.9428
	Correlation parameters	R^2^ = 0.9636 RSS = 160.63
Freundlich	Isotherm parameters	k_F_ (mg^(1−1/nF)^·L^−(1/nF)^·g^−1^) = 19.7944 n_F_ (-) = 8.4674
	Correlation parameters	R^2^ = 0.9648 RSS = 346.55
D-R	Isotherm parameters	B_DR_ (mol^2^·J^−2^) = 8.95·10^−7^ q_max_ (mg·g^−1^) = 33.3948
	Thermodynamic parameters	E_DR_ (J·mol^−1^) = 747.53
	Correlation parameters	R^2^ = 0.9481 RSS = 275.94
Temkin	Isotherm parameters	B_T_ (J·mol^−1^) = 5.7916 b_T_ (J·mol^−1^) = 416.52 A_T_ (L·g^−1^) = 23.6462
	Correlation parameters	R^2^ = 0.9441 RSS = 273.18
Sips	Isotherm parameters	n_S_ (-) = 4.2882 k_S_ (L^(1/nS)^·mol^−(1/nS)^) = 0.2408 q_e,S_ (mg·g^−1^)= 108.54
	Correlation parameters	R^2^ = 0.9787 RSS = 65.87

**Table 2:** Physical-chemical parameters of CCNC real wastewater.

Parameter	Units	Values
[Cr(VI)]_sol_	(mg·L^−1^)	0.450 ± 2.8·10^−3^
COD_sol_	(mg O_2_·L^−1^)	82.67 ± 4.73
pH		7.50 ± 0.21
EC	(mS·cm^−1^)	8.88 ± 0.81

**Table 3:** Comparison of hexavalent adsorption treatments with different types of adsorbents.

Adsorbent	Contact Time (min)	Adsorbent Dosage (mg·L^−1^)	Initial [Cr(VI)] (mg·L^−1^)	pH	q_max_ (mg·g^−1^)	Maximum Removal Yield (%)	Ref.
EPTMAC-modified CNC	60	1000	25	2.5	22.99	96.0	[55]
Black wattle tannin-immobilized CNC	300	500	150	2	104.59		[52]
Poly(m-phenylenediamine)-modified BC nanoparticles	240	400	500	3	434.78		[18]
Carboxymethyl NC-stabilized nZVI	180	300	15	2–3	87.71	100	[62]
Polypyrrole-modified CNC	60	500	10	2	12.67	80	[49]
Hydrophobized CNF	330	500	50	3	70.38	>97.14	[20]
Humic acid-Fe(II) system structured on V, Ti-bearing magnetite surface	700	100	10	2	3.67	90	[45]
Rice husk powder	60	2500	25	6	1.4	87.12	[43]
Lignocellulosic substrate extracted from wheat bran	1440	8000	20	2.5	35		[63]
Activated carbon synthetized from *Z. jujuba*	360	1000	100	2	62	49.6	[64]
CCNC	5	100	70	3	44.36	100	This work

## Data Availability

Not applicable.

## Appendix A

**Table A1:** Adjusted kinetic and correlation parameters of different kinetic models fitted to the experimental data of variable pH (3–9) adsorption of hexavalent chromium onto CCNC.

Kinetic Model	pH Value	(-)	3	7	9
PFO	Kinetic parameters	*k*_1_ (h^−1^)	10.56	0.1222	1.8968
	Correlation parameters	R^2^	0.9956	0.6173	0.7429
		RSS	2.39·10^−2^	6.8179	4.4506
PSO	Kinetic parameters	*k*_2_ (mg·g^−1^·h^−1^)	62.41	1.9935	56.24
		q_e_ (mg·g^−1^)	1.1703	0.7838	0.5121
	Correlation parameters	R^2^	0.9505	0.7197	0.8884
		RSS	0.1998	3.3144	3.3371
Elovich	Kinetic parameters	α (h·mg·g^−1^)	447.99	4481.87	25,757.06
		β (g·mg^−1^)	8.6881	21.83	29.07
	Correlation parameters	R^2^	0.8505	0.7705	0.9152
		RSS	0.4279	2.5517	3.2042
IP	Kinetic parameters: Step 1	k_i,1_ (mg·g^−1^·min^−0.5^)	2.2945	8.21·10^−2^	3.8679
		C_i,1_ (mg·g^−1^)	2.93·10^−2^	0.3628	−0.1093
	Correlation parameters	R^2^	0.9990	0.9633	0.9999
		RSS	2.13·10^−10^	6.23·10^−6^	4.29·10^−5^
	Kinetic parameters: Step 2	k_i,2_ (mg·g^−1^·min^−0.5^)	0	4.98·10^−3^	7.08·10^−2^
		C_i,2_ (mg·g^−1^)	1.1700	0.7313	0.3785
	Correlation parameters	R^2^	0.9999	0.9999	0.9111
		RSS	6.23·10^−6^	4.29·10^−5^	5.36·10^−5^
	Kinetic parameters: Step 3	k_i,3_ (mg·g^−1^·min^−0.5^)			0
		C_i,3_ (mg·g^−1^)			0.5119
	Correlation parameters	R^2^			0.9999
		RSS			6.74·10^−9^

## Appendix B

**Table A2:** Adjusted kinetic and correlation parameters of different kinetic models fitted to the experimental data of variable adsorbent dosage (10–400 mg·L^−1^) adsorption of hexavalent chromium onto CCNC.

Kinetic Model	Adsorbent Dosage	(mg·L^−1^)	10	40	100	400
PFO	Kinetic parameters	*k*_1_ (h^−1^)	1.1327	4.7052	10.56	3.5902
	Correlation parameters	R^2^	0.9772	0.9967	0.9953	0.8034
		RSS	23.23	2.7917	0.2356	2.60·10^−2^
PSO	Kinetic parameters	*k*_2_ (mg·g^−1^·h^−1^)	0.3502	4.2168	36.87	118.33
		q_e_ (mg·g^−1^)	10.01	2.8547	1.1763	0.2821
	Correlation parameters	R^2^	0.9807	0.9907	0.9756	0.9352
		RSS	4.4500	0.1818	8.84·10^−2^	1.02·10^−2^
Elovich	Kinetic parameters	α (h·mg·g^−1^)	184.91	122.09	349.45	193,123
		β (g·mg^−1^)	0.7049	2.3469	7.1582	62.50
	Correlation parameters	R^2^	0.9813	0.9419	0.8505	0.9538
		RSS	1.9938	0.5943	0.1922	6.57·10^−4^
IP	Kinetic parameters: Step 1	k_i,1_ (mg·g^−1^·min^−0.5^)	5.6060	3.5932	2.2945	9.59·10^−2^
		C_i,1_ (mg·g^−1^)	1.3471	6.38·10^−2^	2.93·10^−2^	0.1849
	Correlation parameters	R^2^	0.9939	0.9999	0.9999	0.9968
		RSS	9.16·10^−2^	8.33·10^−9^	1.76·10^−9^	1.19·10^−2^
	Kinetic parameters: Step 2	k_i,2_ (mg·g^−1^·min^−0.5^)	2.6813	0.8322	0	−3·10^−16^
		C_i,2_ (mg·g^−1^)	4.1438	1.9710	1.1700	0.2803
	Correlation parameters	R^2^	0.9999	0.9999	0.9968	0.9999
		RSS	8.33·10^−9^	1.76·10^−9^	1.19·10^−2^	4.99·10^−10^
	Kinetic parameters: Step 3	k_i,3_ (mg·g^−1^·min^−0.5^)	0	0		
		C_i,3_ (mg·g^−1^)	9.5065	2.8032		
	Correlation parameters	R^2^	0.9999	0.9968		
		RSS	1.76·10^−9^	1.19·10^−2^		

## Appendix C

**Table A3:** Adjusted kinetic and correlation parameters of different kinetic models fitted to the experimental data of initial chromium concentration (0.1–10 mg·L^−1^) adsorption of hexavalent chromium onto CCNC.

Kinetic Model	Initial [Cr(VI)]	(mg·L^−1^)	0.1	1	5	10
PFO	Kinetic parameters	*k*_1_ (h^−1^)	10.56	3.3068	0.6412	0.3066
	Correlation parameters	R^2^	0.9953	0.9840	0.8336	0.7091
		RSS	2.39·10^−2^	5.5219	251.84	1464.97
PSO	Kinetic parameters	*k*_2_ (mg·g^−1^·h^−1^)	47.28	1.3056	0.2626	0.1014
		q_e_ (mg·g^−1^)	1.1711	10.37	21.11	34.59
	Correlation parameters	R^2^	0.9638	0.9886	0.9544	0.8993
		RSS	0.1335	2.6428	33.97	227.94
Elovich	Kinetic parameters	α (h·mg·g^−1^)	46.73	5002	28,073	109,044
		β (g·mg^−1^)	3.2292	0.9992	0.5889	0.4108
	Correlation parameters	R^2^	0.7859	0.8232	0.9370	0.9650
		RSS	2.7155	9.8123	7.3288	9.0262
IP	Kinetic parameters: Step 1	k_i,1_ (mg·g^−1^·min^−0.5^)	2.2945	16.61	3.4953	2.6270
		C_i,1_ (mg·g^−1^)	2.93·10^−2^	−0.7973	11.18	20.58
	Correlation parameters	R^2^	0.9995	1.0000	1.0000	0.9284
		RSS	3.49·10^−4^	6.79·10^−10^	1.05·10^−7^	9.67·10^−2^
	Kinetic parameters: Step 2	k_i,2_ (mg·g^−1^·min^−0.5^)	0	4.8746	14.98	16.844
		C_i,2_ (mg·g^−1^)	1.17	5.2377	3.0577	10.66
	Correlation parameters	R^2^	0.9999	0.9800	1.0000	1.0000
		RSS	3.68·10^−9^	0.1236	7.21·10^−8^	4.16·10^−7^
	Kinetic parameters: Step 3	k_i,3_ (mg·g^−1^·min^−0.5^)		6.79·10^−2^	0.6576	1.7653
		C_i,3_ (mg·g^−1^)		10.04	17.93	25.60
	Correlation parameters	R^2^		0.698	0.9985	0.9990
		RSS		3.98·10^−2^	0.8894	5.36·10^−2^

**Table A4:** Adjusted kinetic and correlation parameters of different kinetic models fitted to the experimental data of initial chromium concentration (25-70 mg·L^−1^) adsorption of hexavalent chromium onto CCNC (cont.).

Kinetic Model	Initial [Cr(VI)]	(mg·L^−1^)	25	50	70
PFO	Kinetic parameters	*k*_1_ (h^−1^)	0.2233	0.3162	0.4961
	Correlation parameters	R^2^	0.8524	0.6837	0.7066
		RSS	707.21	1407.73	1969.33
PSO	Kinetic parameters	*k*_2_ (mg·g^−1^·h^−1^)	38.81	36.74	44.77
		q_e_ (mg·g^−1^)	3.22·10^−2^	9.32·10^−2^	6.41·10^−2^
	Correlation parameters	R^2^	0.9507	0.9549	0.8838
		RSS	109.16	98.19	471.17
Elovich	Kinetic parameters	α (h·mg·g^−1^)	594	570,868	150947
		β (g·mg^−1^)	0.2177	0.4309	0.3233
	Correlation parameters	R^2^	0.7859	0.8232	0.9370
		RSS	2.7086	27.24	295.94
IP	Kinetic parameters: Step 1	k_i,1_ (mg·g^−1^·min^−0.5^)	35.31	19.42	34.60
		C_i,1_ (mg·g^−1^)	−3.0290	14.96	13.90
	Correlation parameters	R^2^	1.0000	1.0000	1.0000
		RSS	7.65·10^−8^	5.82·10^−9^	1.45·10^−7^
	Kinetic parameters: Step 2	k_i,2_ (mg·g^−1^·min^−0.5^)	0	0	2.8476
		C_i,2_ (mg·g^−1^)	18.31	27.09	30.11
	Correlation parameters	R^2^	0.9999	0.9999	0.9890
		RSS	1.41·10^−10^	3.18·10^−9^	2.3781
	Kinetic parameters: Step 3	k_i,3_ (mg·g^−1^·min^−0.5^)	4.0010	1.9211	
		C_i,3_ (mg·g^−1^)	18.31	27.09	
	Correlation parameters	R^2^	0.9985	0.9950	
		RSS	0.4068	0.3077	

## Appendix D

**Table A5:** Adjusted kinetic and correlation parameters of different kinetic models fitted to the experimental data of variable temperature during adsorption of hexavalent chromium onto CCNC and Arrhenius equation parameters and thermodynamic calculations.

Kinetic Model	Temperature	(°C)	17	30	42	56
PSO	Kinetic parameters	*k*_2_ (mg·g^−1^·h^−1^)	0.9243	2.5799	5.3297	8.4657
		q_e_ (mg·g^−1^)	10.63	10.45	10.38	10.36
	Correlation parameters	R^2^	0.9961	0.9872	0.9939	0.9997
		RSS	0.7617	2.3274	1.0576	6.13·10^−2^
Arrhenius equation	**Parameters**		**Values**			
	Activation Energy Preexponential factor	E_A_ (J·mol^−1^)	45,447			
		*k*_0_ (mg·g^−1^·h^−1^)	1.59·10^8^			
	Correlation parameters	R^2^	0.9779			
		RSS	6.18·10^−2^			

## Appendix E

**Table A6:** Adjusted kinetic and correlation parameters of different kinetic models fitted to the experimental data of hexavalent chromium adsorption on real wastewater treatment.

Kinetic Model			Real Wastewater Treatment
Pseudo-first order	Kinetic parameters	k_1_ (h^−1^)	2.9262
	Correlation parameters	R^2^	0.9920
		RSS	0.4067
PSO	Kinetic parameters	k_2_ (mg·g^−1^·h^−1^)	1.1405
		q_e_ (mg·g^−1^)	4.1621
	Correlation parameters	R^2^	0.9897
		RSS	0.3201
Elovich	Kinetic parameters	α (h·mg·g^−1^)	67.42
		β (g·mg^−1^)	1.3789
	Correlation parameters	R^2^	0.9730
		RSS	0.4487
IP	Kinetic parameters: Step 1	k_i,1_ (mg·g^−1^·min^−0.5^)	3.4863
		C_i,1_ (mg·g^−1^)	0.1324
	Correlation parameters	R^2^	0.9983
		RSS	5.03·10^−2^
	Kinetic parameters: Step 2	k_i,2_ (mg·g^−1^·min^−0.5^)	0.9718
		C_i,2_ (mg·g^−1^)	2.5269
	Correlation parameters	R^2^	0.9999
		RSS	5.36·10^−11^
	Kinetic parameters: Step 3	k_i,3_ (mg·g^−1^·min^−0.5^)	0.1801
		C_i,3_ (mg·g^−1^)	3.4965
	Correlation parameters	R^2^	0.9999
		RSS	1.20·10^−9^
